# Keloids and inflammation: the crucial role of IL-33 in epidermal changes

**DOI:** 10.3389/fimmu.2025.1514618

**Published:** 2025-03-31

**Authors:** ZongAn Chen, YaTing Yang, XiuXia Wang, LingLing Xia, WenBo Wang, XiaoLi Wu, Zhen Gao

**Affiliations:** Department of Plastic and Reconstructive Surgery, Shanghai Ninth People’s Hospital, Shanghai Jiao Tong University School of Medicine, Shanghai, China

**Keywords:** keloid, keratinocyte, inflammation, IL-33, epidermis

## Abstract

**Introduction:**

Keloids are benign fibroproliferative disorders characterized by excessive collagen deposition and inflammation that extend beyond the original wound boundaries. IL-33 is an alarmin cytokine released upon cellular damage or stress. Dysregulation of IL-33 in epidermal keratinocytes compromises the skin barrier and triggers chronic inflammation.

**Method:**

In this study, we first noticed an increased expression of IL-33 in the keratinocytes of keloid epidermis through histological staining. Then, an increased expression of IL-33 receptor (ST2) in the lymphocytes infiltrating the superficial dermis of keloid scars were identified through histological staining and flow cytometry analysis. The IFN-γ-IL-33 loop between lymphocytes and keratinocytes were further revealed by flow cytometry and Western blotting analysis. The abnormal keratinocyte differentiation in epiderm is mediated by IFN-γ-IL-33 loop were confirmed by *in vitro* studies in HaCaT cells via Western blotting analysis and immunofluorescence staining. Finally, the IFN-γ-IL-33 loop were also verified in cocultured peripheral blood mononuclear cells and HaCaT through ELISA analysis.

**Results:**

Our results demonstrate that IL-33 levels are significantly elevated in the epidermis of keloid tissues, where it functions as an alarmin, promoting a chronic inflammatory response. We further reveal a feedback loop between IL-33 and interferon-gamma (IFN-γ), whereby IL-33 induces IFN-g production in lymphocytes, which in turn stimulates keratinocytes to produce more IL-33. This loop contributes to impaired keratinocyte differentiation and skin barrier dysfunction, exacerbating the inflammatory environment.

**Discussion:**

By elucidating the role of the IL-33/ST2 axis in keloid formation, this research provides valuable insights into potential therapeutic targets for managing this challenging condition.

## Graphical Abstract

The IL-33/ST2 axis between keloid keratinocytes and T cells.

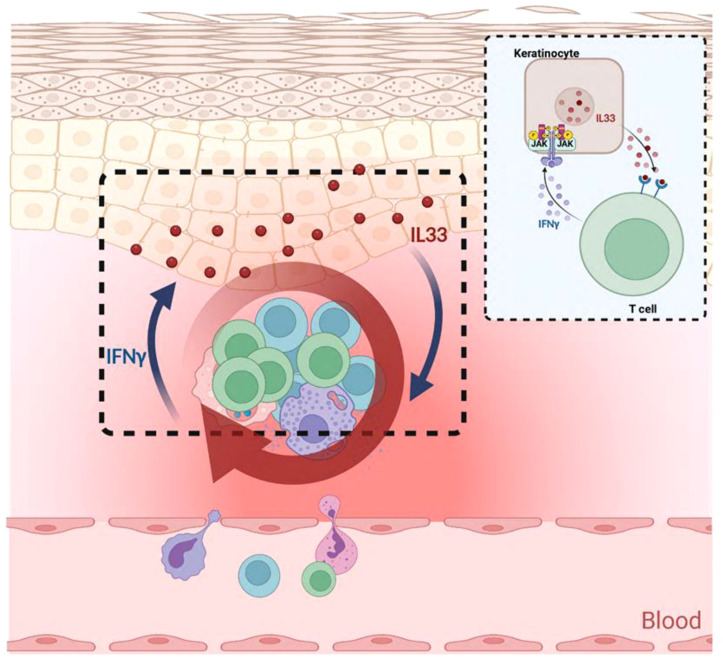

## 1 Introduction

Keloids are benign fibroproliferative disorders that initially manifest as inflammatory nodules and eventually extend beyond the original wound boundaries. These lesions are often associated with symptoms such as itching and pain. The pathogenesis of keloids is complex, involving a combination of inflammation, mechanical tension, and genetic susceptibility (1–4). Inflammatory responses are a hallmark of keloid disease, characterized by the infiltration of immune cells, including macrophages, T cells, and mast cells, into the affected tissue (5). These immune cells release various pro-inflammatory cytokines and growth factors, such as IL-1, IL-6, TNF-α, and TGF-β, which drive the chronic inflammatory environment and promote excessive collagen deposition (6, 7).

IL-33 is a member of the IL-1 cytokine family and functions as an alarmin, released upon cellular damage or stress. It is predominantly expressed in epithelial and endothelial cells, where it plays a pivotal role in initiating and amplifying inflammatory responses (8, 9). Its receptor, Suppression of Tumorigenicity 2 (ST2), is expressed on various immune cells, including Th2 cells, mast cells, and macrophages, leading to the release of additional pro-inflammatory cytokines such as IL-1, IL-6, and TNF-α (10). Recent studies have highlighted the critical role of IL-33 in the progression of skin inflammation and fibrosis (11, 12). Dysregulation of IL-33 in epidermal keratinocytes compromises the skin barrier and triggers chronic inflammation (13, 14). This aligns with observations of epidermal abnormalities and infiltration of inflammatory cells in keloids (5, 15).

In this study, we identified that IL-33 expression is markedly elevated in the epidermis of keloids, with keratinocytes being the major source of IL-33. Additionally, ST2 expression was increased on the infiltrating inflammatory cells in keloid tissue. Furthermore, we discovered an IFN-γ-IL-33 loop between lymphocytes and keratinocytes that amplifies inflammatory responses and impairs keratinocyte differentiation. Elucidating the role of IL-33 in keloids provides novel insights into keloid formation and progression, as well as potential therapeutic targets.

## 2 Method

### 2.1 Patients and specimen collection

All human specimens were collected during plastic surgery at Shanghai Ninth People’s Hospital, Shanghai Jiao Tong University School of Medicine. Patients with keloid scars met the following criteria: (i) a diagnosis confirmed by at least three clinicians, (ii) the keloid scar had persisted for over one year, and (iii) no steroid treatment had been administered within the past year. Patients with mature scars were excluded if they had skin diseases, such as psoriasis or atopic dermatitis, or other immunopathologies.

This study was approved by the Clinical Research Ethics Board of Shanghai Ninth People’s Hospital, and written informed consent was obtained from all participants. The clinical characteristics of the volunteers are detailed in **Supplementary Table 1**.

### 2.2 Chemical reagents

IFN-γ (#300-02) and IL-33 (#200-33) were purchased from PeproTech, Inc. (U.S.A). Itacitinib (INCB39110), Fedratinib (TG101348), and Tofacitinib (S2789) were obtained from Selleck Chemicals (China). Plasmids containing shRNA targeting of IL-33 were purchased from GenePharma (Shanghai, China).

### 2.3 Sample preparation

Both blood and tissue samples were processed within 4 hours of collection. Human peripheral blood mononuclear cells (PBMCs) were prepared using Ficoll-Hypaque gradient centrifugation, following the manufacturer’s instructions. Fresh PBMCs were incubated overnight with the patients’ serum in a 5% CO_2_ incubator before treatment.

Skin samples were stored at 4°C in 50-mL sterile tubes containing phosphate-buffered saline (PBS) immediately after excision. To isolate lymphocytes from keloid scars, the keloid tissues were treated with collagenase NB4 (SERVA Electrophoresis, Heidelberg, Germany) dissolved in Dulbecco’s Modified Eagle’s Medium (DMEM, HyClone, Logan, UT, USA, 0.25% v/v) overnight a 37°C in a 5% CO_2_ incubator. After digestion, cells were harvested by sequential filtration through 100-μm and 40-μm strainers. The cells were then centrifuged and washed twice in PBS containing 2% fetal bovine serum (FBS) (Gibco-Invitrogen Corp., Grand Island, NY, USA) before further treatment.

### 2.4 Cell culture and plasmid transfection

HaCaT cells were purchased from the National Collection of Authenticated Cell Cultures (China) and cultured in DMEM supplemented with 10% FBS and 1% penicillin/streptomycin (Gibco). PBMCs collected from keloid patients were cultured in Roswell Park Memorial Institute (RPMI) 1640 medium with 10% FBS and 1% penicillin/streptomycin.

Short hairpin RNA (shRNA) was used to knock down IL-33 in HaCaT cells. After 48 hours of transfection with Lipofectamine™ 3000 (2 μL/μg DNA), following the manufacturer’s instructions, the cells were harvested and screened with neomycin for one week before further experimentation.

### 2.5 Immunohistochemical and Immunofluorescence

To prepare specimens for staining, skin samples were fixed in 4% paraformaldehyde at 4°C overnight. The specimens were then embedded in paraffin and sectioned into 5-μm-thick slices. The staining process was performed as previously described (16). Briefly, deparaffinized sections underwent heat-mediated antigen retrieval. Sections of keloid and mature scars were incubated with the primary antibodies, followed by the appropriate conjugated secondary antibodies, as listed in **Supplementary Table 2**. For IHC, 3,3’-diaminobenzidine (DAB, G1211, Servicebio, Wuhan, China) was used as a chromogen to visualize the bound antibodies, and the slides were counterstained with hematoxylin. Cell count was conducted using ImageJ 1.53c software (NIH, U.S.A.) in five randomly selected fields (20× magnification) under a microscope for semi-quantification.

HaCaT cells were treated with or without Itacitinib (1.0 nM) and shRNA in the presence of 50 ng/mL IFN-γ for 48 hours before immunofluorescence (IF) staining. The antibodies used in this study are listed in **Supplementary Table 2**.

### 2.6 Flow cytometry analysis

Flow cytometry analysis was performed as previously described (17).Briefly, PBMCs were resuspended in Flow Cytometry Staining Buffer (BD Biosciences) and adjusted to a concentration of 2 × 10^7^ cells/mL. Fifty microliters of the cell suspension were incubated with 50 μL of antibody cocktail for at least 60 minutes at 2–8°C. The cells were then washed and resuspended in buffer for flow cytometry analysis.

For the detection of intracellular cytokine production, PBMCs were stimulated with 100 ng/mL IL-33 for 24 hours prior to flow cytometry analysis. The intracellular cytokine staining procedure was similar to that for the surface markers, where cells were incubated with primary antibodies in staining buffer (eBioscience), followed by fixation and permeabilization for intracellular and intranuclear staining.

Data were acquired using an LSRFortessa flow cytometer (BD Biosciences, U.S.A.) and analyzed with FlowJo software (Tree Star Inc., U.S.A.). The antibodies used in this study are listed in **Supplementary Table 3**, and the gating strategies are shown in **Supplementary Figure 1**.

### 2.7 Western blotting analysis

HaCaT cells were treated with or without IFN-γ (0, 5, 10, 15, 50, and 100 ng/mL), Itacitinib (1.0 nM), Fedratinib (1.0 nM), and Tofacitinib (0.5 nM) for 48 hours before Western blot (WB) analysis. Protein was extracted and quantified as previously described (16). The antibodies used in this study are listed in **Supplementary Table 2**. Immunoreactive bands were detected using chemiluminescence reagents, and standardized integrated density of bands was quantified using ImageJ 1.53c software (NIH, U.S.A.) for triplicate samples.

### 2.8 ELISA analysis

A transwell system was used for cell co-culture, with 0.2 × 10^6^ HaCaT cells and 0.5 × 10^6^ PBMCs seeded in each well of a 24-well plate. HaCaT cells were pretreated with 50 ng/mL IFN-γ for 24 hours before being co-cultured with untreated PBMCs. Similarly, PBMCs were pretreated with 100 ng/mL IL-33 for 24 hours before being co-cultured with untreated HaCaT cells. After 48 hours of co-culture, the released IL-33 (EK133-A13340321) and IFN-γ (EK180-A18040624) proteins were measured using ELISA kits (Multi Sciences (Lianke) Biotech Co., Ltd, China) according to the manufacturer’s instructions, with absorbance measured at a wavelength of 450 nm. All assays were performed in triplicate.

### 2.9 Statistical analysis

Statistical significance was determined using GraphPad Prism 10 software (GraphPad Software, USA). A Student’s *t*-test was used and differences were deemed significant when P < 0.05.

## 3 Result

### 3.1 Increased expression of IL-33 in the keratinocytes of keloid epidermis

**Figure 1:**
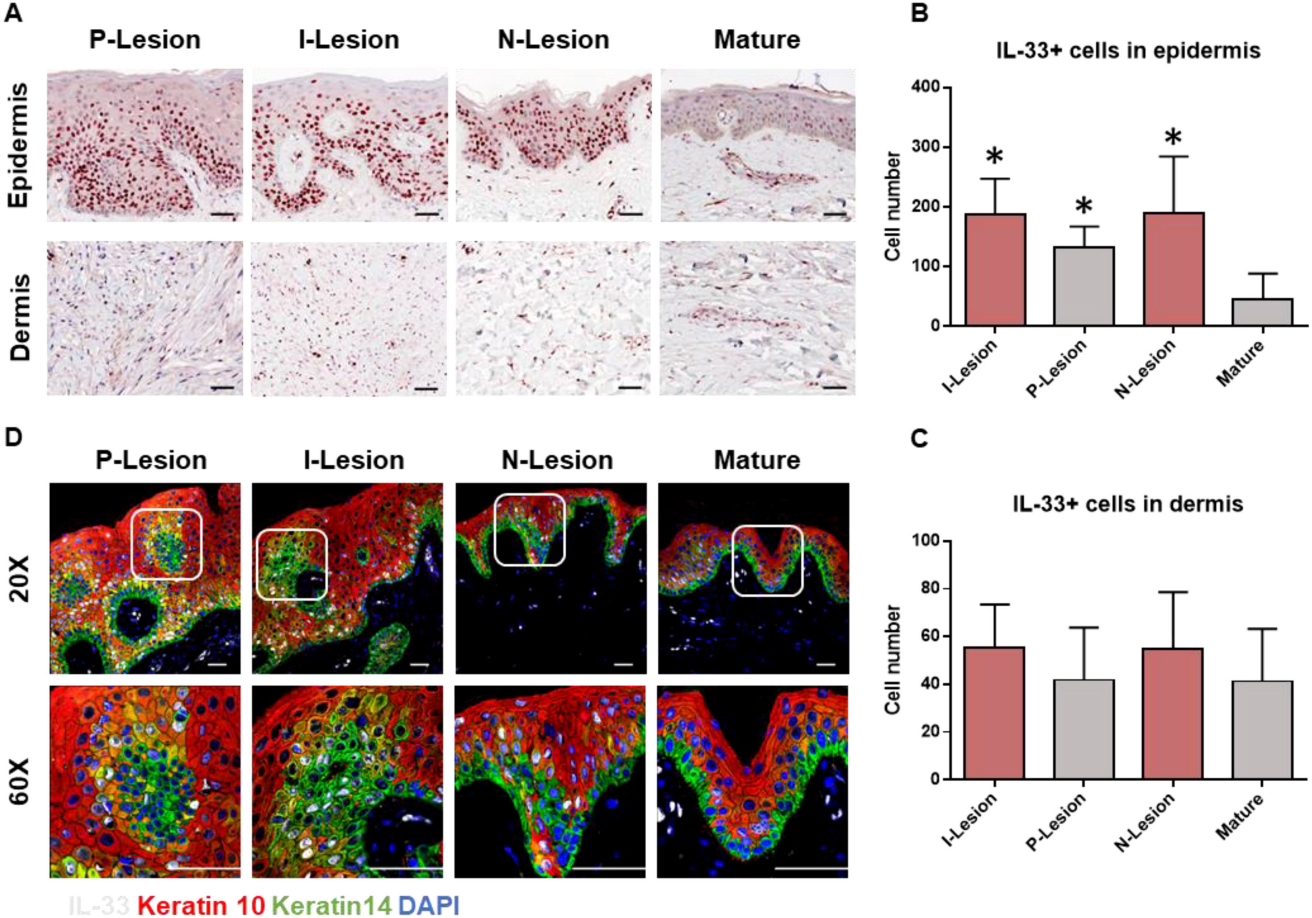
Increased expression of IL-33 in the epidermis of keloid scar. **(A)** IHC analysis of IL-33 in the epidermis and dermis of mature scars and various regions of keloid scars, including the peripheral (P-Lesion), interior (I-Lesion), and non-lesioned (N-Lesion) skin. **(B)** Quantitative analysis of IL-33+ cells in the epidermis of mature scars and different keloid scar regions. **(C)** Quantitative analysis of IL-33+ cells in the dermis of mature scars and various keloid scar regions. **(D)** IF analysis demonstrating the colocalization of IL-33(white) with K14+ (green) and K10+ (red) keratinocytes (IL-33 is shown in white). *Statistical significance is indicated as P < 0.05 compared to the mature scar group.

Chronic skin inflammation is a key pathological feature of keloid formation and progression. In this study, we investigated the distribution patterns and characteristics of epithelial-derived alarmins (IL-25, IL-33, and TSLP) in keloid lesions using IHC staining. **Figures 1A, B** show that IL-33 expression was significantly upregulated in both the peripheral (P-Lesion) and interior (I-Lesion) regions of keloid lesions, as well as in non-lesioned (N-Lesion) skin from keloid patients, compared to mature scars. However, IL-33 enrichment was not observed in the dermis of keloid scars, surrounding normal skin, or mature scars (**Figures 1A, C**). IL-25 and TSLP expression did not show significant differences in mature scars or various keloid regions (**Supplementary Figure 2**). The WB results also revealed an elevated level of both cleaved IL-33 and full-length IL-33 in keloid tissues compared to mature scars (**Supplementary Figure 3**). IF staining further revealed that IL-33 (white) was localized in K14+ (green) and K10+ (red) keratinocytes (**Figure 1D**).

### 3.2 Increased expression of IL-33 receptor (ST2) in the lymphocytes infiltrating the superficial dermis of keloid scars

**Figure 2:**
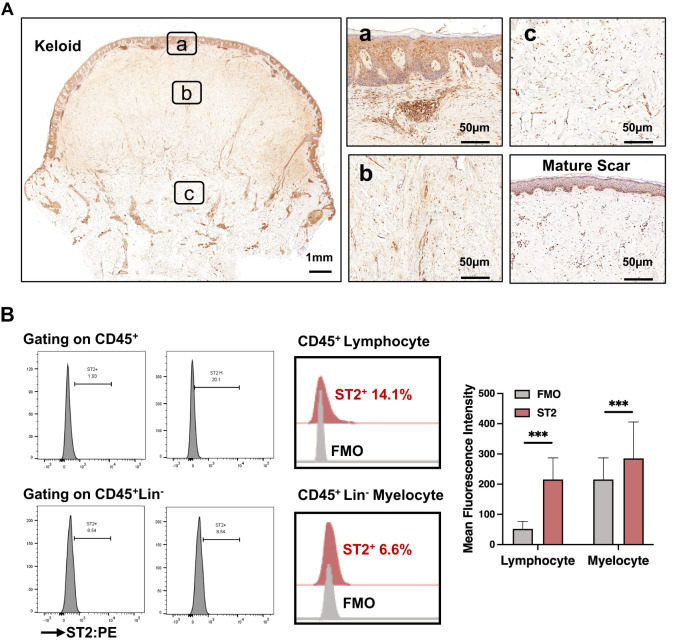
Increased expression of IL-33 receptor (ST2) in the lymphocytes infiltrating the superficial dermis of keloid scars. **(A)** IHC analysis of ST2 expression in the epidermis and dermis of mature scars and various regions of keloid scars, including the peripheral (P-Lesion), interior (I-Lesion), and non-lesioned (N-Lesion) skin. **(B)** Flow cytometric analysis of ST2+ skin cells isolated from keloid tissue. *** Statistical significance is indicated as P < 0.001.

We analyzed ST2 expression in keloid and mature scars using IHC staining. As shown in **Figure 2A**; **Supplementary Figure 4**, ST2 expression was significantly increased in the superficial dermis of keloid scars compared to mature scars. However, ST2 expression in the dermis and epidermis did not differ significantly across scar types (**Supplementary Figure 4**). Flow cytometric analysis of ST2-positive cells from keloid tissue revealed that CD45+ lymphocytes and CD45+Lin- myelocytes were the primary ST2-expressing cells, with positive rates of 14.1 ± 6.6% and 6.6 ± 3.5%, respectively (**Figure 2B**). Among CD45+ST2+ lymphocytes, 44.9 ± 12.8% were CD19-CD3+CD4+ T cells, 25.8 ± 10.5% were CD19-CD3+CD8+ T cells, 6.8 ± 3% were CD19+CD56+ NK cells, and 2.3 ± 2.4% were CD19+ B cells (**Table 1**). These findings indicate that increased ST2 expression in the superficial dermis of keloid scars is primarily associated with lymphocytes, particularly CD4+ T cells.

**Table 1:** Subsets of ST2^+^ lymphocytes among CD45+ cells in keloid.

Sample ID	ST2^+^	ST2^+^	Subsets of ST2+ lymphocytes(%CD45^+^ST2^+^)				
	% Myelocyte	%Lymphocyte	CD3^+^ T	CD4^+^ T	CD8^+^ T	NK	CD19^+^ B
K1	9.5%	23.5%	58.8%	25.8%	21.3%	14.9%	5.6%
K2	10.3%	14.3%	69.4%	41.8%	17.6%	4.7%	8.5%
K3	8.5%	20.1%	77.7%	39.9%	26.0%	6.1%	0.6%
K4	4.1%	9.4%	78.5%	55.0%	18.0%	6.1%	1.4%
K5	8.9%	21.0%	84.0%	51.6%	23.5%	4.7%	0.8%
K6	3.2%	8.4%	73.4%	47.9%	17.7%	9.8%	1.9%
K7	2.9%	6.8%	71.0%	21.2%	38.7%	7.0%	3.0%
K8	2.6%	4.3%	87.5%	31.0%	52.0%	3.0%	0.0%
K9	6.3%	6.3%	79.1%	47.1%	24.6%	6.2%	2.3%
K10	13.9%	20.7%	83.0%	53.5%	21.7%	8.4%	0.3%
K11	7.4%	16.5%	87.1%	67.3%	13.4%	5.1%	3.2%
K12	3.1%	12.2%	87.5%	47.2%	34.1%	5.2%	0.7%
K13	4.7%	19.9%	83.4%	54.0%	26.2%	6.7%	1.1%
Mean	6.6%	14.1%	78.5%	44.9%	25.8%	6.8%	2.3%
SD	3.5%	6.6%	8.5%	12.8%	10.5%	3.0%	2.4%

### 3.3 The IFN-γ-IL-33 loop between lymphocytes and keratinocytes

**Figure 3:**
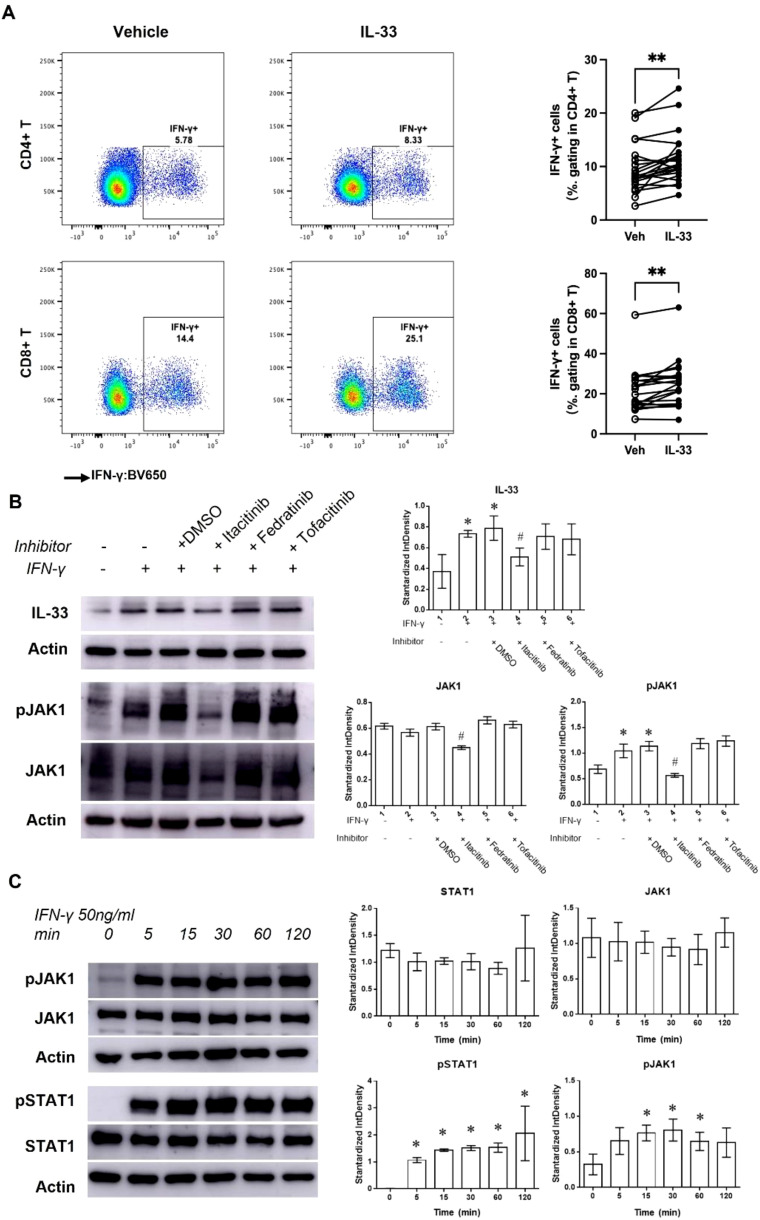
The IFN-γ-IL-33 loop between lymphocytes and keratinocytes. **(A)** Flow cytometric analysis of IFN-γ+ PBMCs pretreated with 100 ng/mL IL-33 for 24 hours. **Statistical significance is indicated as P < 0.01. **(B)** Western blot analysis and quantification of IL-33, pJAK1, and JAK1 levels in HaCaT cells treated with or without JAK inhibitors (Itacitinib [1 nM], Fedratinib [1 nM], and Tofacitinib [0.5 nM]) in the presence of 50 ng/mL IFN-γ for 48 hours. **(C)** Western blot analysis and quantification of pJAK1, JAK1, pSTAT1, and STAT1 levels in keratinocytes treated with 50 ng/mL IFN-γ for various durations. *Statistical significance is indicated as follows*: * P < 0.05 compared to cells treated without IFN-γ or inhibitors, ^#^ P < 0.05 compared to cells treated with IFN-γ alone.

IL-33 significantly increased IFN-γ expression in CD4+ (from 5.78% to 7.33%) and CD8+ (from 14.4% to 25.1%) PBMCs (**Figure 3A**), while IL-4 and IL-17 expression remained unchanged (**Supplementary Figure 5**). This suggests that IL-33 secreted by keratinocytes induces IFN-γ production in lymphocytes. Conversely, treating HaCaT cells with IFN-γ promoted IL-33 production, with maximum effects at 50 ng/mL (**Supplementary Figure 6A**), forming an IFN-γ-IL-33 feedback loop.

IFN-γ plays a crucial role in immune responses by activating JAK/STAT pathway (18). Western blot (WB) analysis showed that IFN-γ treatment (50 ng/mL for 48 hours) significantly increased IL-33, pJAK1, and pSTAT1 levels, without affecting JAK1 or STAT1 (**Figure 3C**). Treatment with the JAK1 inhibitor Itacitinib reduced pJAK1 and IL-33 levels under IFN-γ stimulation, confirming JAK1/STAT1 pathway involvement. Further studies showed that IFN-γ-induced JAK1/STAT1 activation began within 5 minutes and persisted for over 120 minutes (**Figure 3B**).

We also observed increased pJAK2 levels 30 minutes post-IFN-γ treatment (**Supplementary Figure 6B**), but JAK2 and JAK3 inhibitors (Fedratinib and Tofacitinib) did not affect the JAK1/STAT1 pathway or IL-33 levels (**Figure 3B**). These findings confirm that the IFN-γ-IL-33 loop operates through the JAK1/STAT1 pathway.

### 3.4 IFN-γ-IL-33 loop mediated abnormal keratinocyte differentiation in epidermis

**Figure 4:**
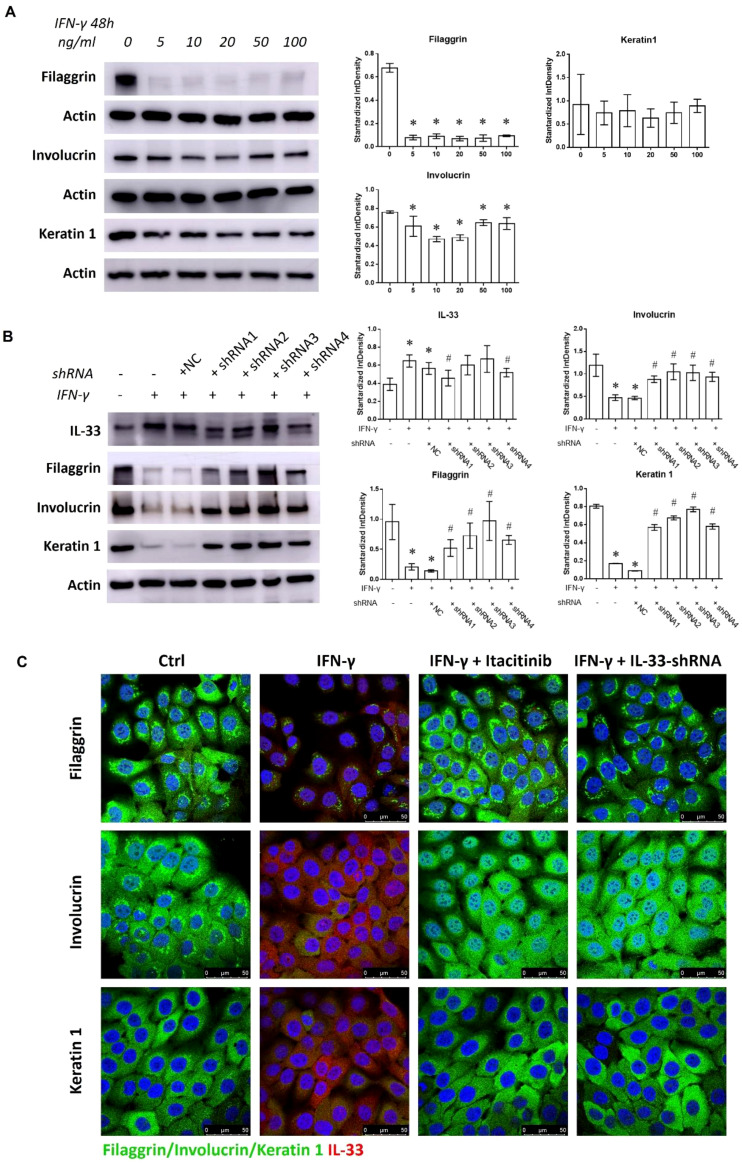
Discouraged keratinocyte differentiation mediated by the IFN-γ-IL-33 loop. **(A)** Western blot analysis of Filaggrin, Involucrin, and Keratin 1 levels in HaCaT cells treated with varying concentrations of IFN-γ (0, 5, 10, 20, 50, and 100 ng/mL) for 48 hours. **(B)** Western blot analysis of IL-33, Filaggrin, Involucrin, and Keratin 1 levels in HaCaT cells treated with or without shRNA1, shRNA2, shRNA3, and shRNA4 in the presence of 50 ng/mL IFN-γ for 48 hours. **(C)** Immunofluorescence staining of Filaggrin, Involucrin, Keratin 1, and IL-33 in HaCaT cells treated with or without Itacitinib (1 nM) or IL-33 shRNA in the presence of 50 ng/mL IFN-γ for 48 hours. NC refers to the negative control. *Statistical significance is indicated as follows:* * P < 0.05 compared to cells treated without IFN-γ or inhibitors, ^#^ P < 0.05 compared to cells treated with IFN-γ alone.

IL-33 disrupts keratinocyte differentiation, impairing the skin barrier and promoting chronic inflammation (19, 20). HaCaT cells treated with 5 to 100 ng/mL IFN-γ showed reduced Filaggrin and Involucrin expression alongside increased IL-33 production (**Figures 4A**; **Supplementary Figure 6A**). IL-33 knockdown via shRNA restored Filaggrin, Involucrin, and Keratin 1 levels and suppressed JAK1/STAT1 activation in the presence of 50 ng/mL IFN-γ (**Figure 4B**; **Supplementary Figure 6C**). Similarly, immunofluorescence analysis revealed that Itacitinib or IL-33 shRNA treatment restored keratinocyte marker expression in IFN-γ-treated cells (**Figure 4C**). These results indicate that IL-33 enrichment from the IFN-γ-IL-33 loop mediates keratinocyte differentiation defects in the epidermis.

### 3.5 *In vitro* verification of the IFN-γ-IL-33 loop in cocultured peripheral blood mononuclear cells and HaCaT

**Figure 5:**
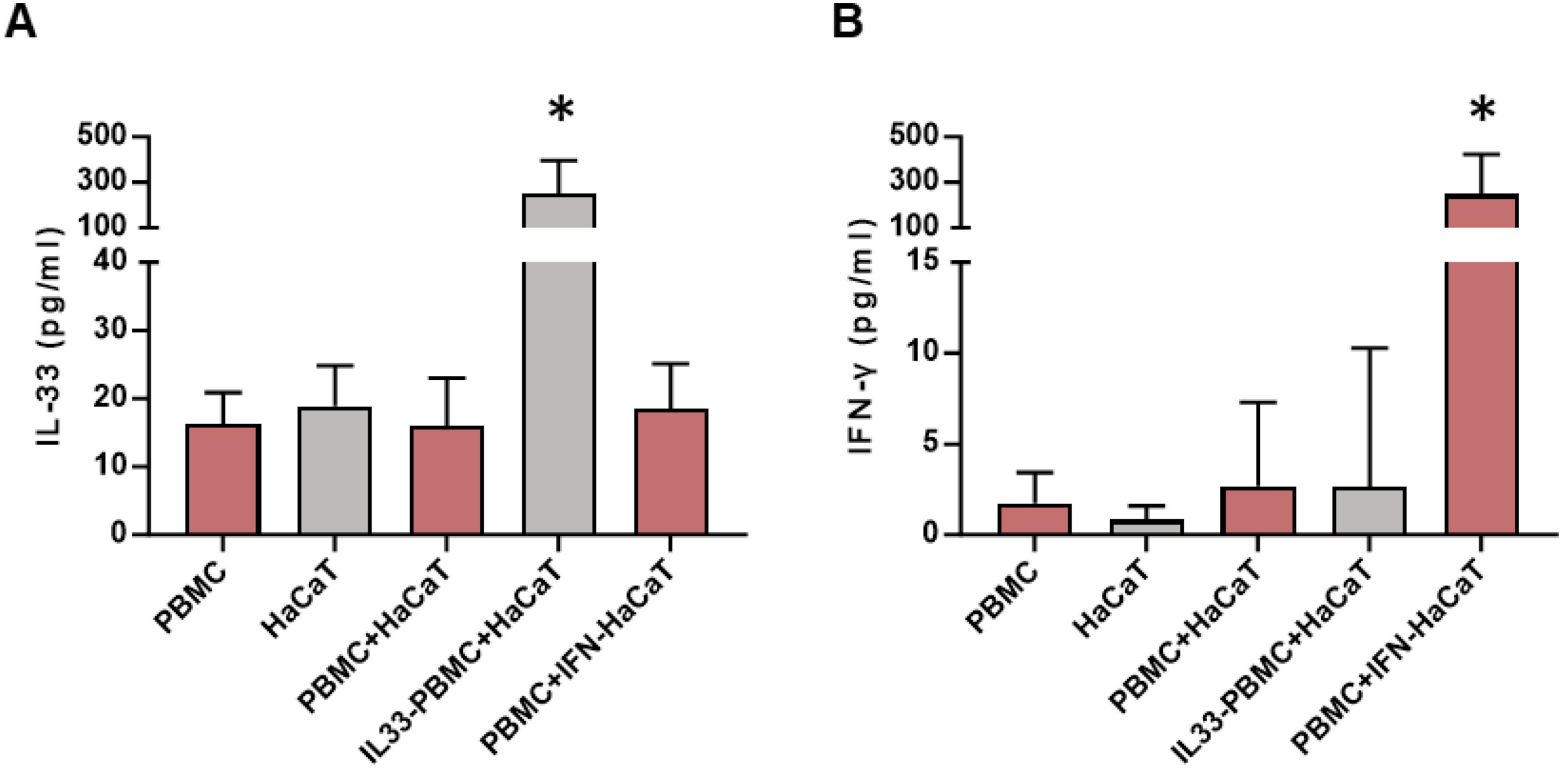
ELISA analysis of IL-33 and IFN-γ concentrations in the co-culture system. **(A)** IL-33 levels in the co-culture media after 48 hours of co-culture. IL33-PBMC refers to PBMCs pre-incubated with 100 ng/mL IL-33 for 24 hours, followed by washing with PBS prior to co-culturing with HaCaT cells. **(B)** IFN-γ levels in the co-culture media after 48 hours of co-culture. IFN-HaCaT refers to HaCaT cells pre-incubated with 50 ng/mL IFN-γ for 24 hours, followed by washing with PBS before co-culturing with PBMCs. * represents P < 0.05 in compared to PBMC group. *Statistical significance is indicated as P < 0.05 compared to the PBMC group.

Lymphocytes residing in the skin originate from circulating PBMCs and communicate with local cells via cytokines and chemokines to maintain skin homeostasis and regulate skin inflammation (21). To verify the IFN-γ-IL-33 loop *in vitro*, PBMCs from keloid patients were co-cultured with HaCaT cells. IL-33 levels remained unchanged when HaCaT cells were cultured alone or co-cultured with unstimulated PBMCs (**Figure 5A**). However, co-culture with PBMCs pretreated with IL-33 significantly increased IL-33 levels, and co-culture with HaCaT cells pretreated with IFN-γ elevated IFN-γ levels in the media (**Figure 5B**). These findings demonstrate that the IFN-γ-IL-33 loop between PBMCs and keratinocytes enhances IFN-γ production in lymphocytes and IL-33 infiltration in the epidermis, contributing to IL-33 accumulation and compromised skin barrier function in keloid skin.

## 4 Discussion

Inflammation in the dermis has traditionally been the focus of keloid research. However, accumulating evidence suggests that abnormalities in the epidermis also play a critical role in skin inflammation. IL-33, primarily stored in the nuclei of endothelial and epithelial cells, is rapidly released upon cellular damage or stress (22). Its receptor, ST2, is expressed by various immune cells involved in both innate immunity (e.g., mast cells and macrophages) and adaptive immunity (e.g., CD4+ and CD8+ T cells) (23, 24). The IL-33/ST2 axis is widely recognized for its role in immunity and tissue homeostasis, promoting wound healing and tissue repair (25). In this study, we identified excessive expression of IL-33 in keratinocytes and ST2 in lymphocytes within keloid scars. Given that increases in IL-33 are frequently observed during inflammatory processes, our findings confirm that the epidermis is also inflamed and damaged during keloid progression (26, 27).

IFN-γ, primarily produced by Th1 cells, serves as a key factor in activating macrophages, enhancing bactericidal activity, and promoting the differentiation of Th1 cells. IL-33, belonging to the IL-1 family, functions as an alarmin involved in inflammation and immune responses, particularly playing a significant role in Th2-type immune responses. Studies have shown that the regulatory effect of IFN-γ on IL-33 is cell-type dependent. In lung fibroblasts, IFN-γ induces the expression of LMP2 (latent membrane protein 2) proteasome through activation of the STAT1 signaling pathway, leading to the degradation of IL-33 protein and significantly downregulating IL-33 mRNA expression (28). However, in skin keratinocytes, IFN-γ promotes the expression of IL-33 mRNA and protein by activating signaling pathways including ERK, p38, EGFR, and JAK in a dose- and time-dependent way (29). Previously, we reported increased T lymphocyte infiltration in keloid tissues (17). Here, we demonstrated that the enhancement of the IL-33/ST2 axis in keloid scars results from an IFN-γ-IL-33 feedback loop between keratinocytes and lymphocytes. Specifically, IL-33 stimulates lymphocytes to produce excess IFN-γ, which, in turn, induces keratinocytes to overexpress IL-33 via the JAK1/STAT1 signaling pathway (30). This reciprocal interaction between the epidermis and immune cells contributes to the establishment and maintenance of the inflammatory microenvironment in keloid skin.

Beyond its role in regulating immune responses, IL-33 also affects the integrity of the skin barrier. Keratinocytes, as the primary component of the epidermis, play a pivotal role in maintaining skin structure and function. Although the functional state of the keloid epidermis remains debated, abnormalities such as increased epidermal thickness and altered keratin expression are well-documented (15). IL-33 influences keratinocyte proliferation and differentiation, affecting the production of key structural proteins, including filaggrin, involucrin, and keratin 1 (25). Filaggrin and involucrin contribute to the formation of the cornified cell envelope, which protects the skin from external microorganisms and prevents water loss (31). Keratin 1 is essential for safeguarding the skin from physical and chemical damage (32). Our study revealed that the IFN-γ-IL-33 loop leads to IL-33 accumulation in the epidermis, impairing the production of these proteins. This disruption compromises the skin barrier, increasing its susceptibility to external inflammatory stimuli.

We propose that epidermal IL-33 levels in keloid patients could serve as a biomarker to assess keloid severity and guide clinical decisions. Additionally, targeting the IL-33/ST2 axis offers a promising therapeutic approach for inflammatory diseases. Recent studies suggest that inhibiting ILC2s through the IL-33/ST2 and JAK/STAT pathways can alleviate type 2 inflammation in OVA-induced allergic rhinitis (33). In our study, blocking IL-33 via shRNA successfully reversed its inhibitory effects on keratinocyte maturation, highlighting the IFN-γ-IL-33 loop as a potential target for keloid treatment.

IL-33 is primarily expressed in the nucleus and is released upon stress-induced necrosis. In tissues, it undergoes enzymatic cleavage to function. Its receptor, ST2, is expressed on cell membrane but can release its extracellular domain as neutralizing antibodies, particularly in high ST2+ environments, enabling interactions with IL-33 in skin tissues. IL-33 acts as an alarmin in various diseases, including inflammatory bowel disease, asthma, and atopic dermatitis (AD) (34, 35). AD, characterized by type 2 skin inflammation, is an independent risk factor for keloid formation (36, 37). IL-33 transgenic mice expressing IL-33 in keratinocytes spontaneously develop AD-like eczema, suggesting that IL-33 alone can drive AD pathogenesis (38). Furthermore, IL-33 reduces skin barrier function by downregulating filaggrin, promoting allergen exposure and inflammation (39).

The IL-33/ST2 axis is also linked to pain and itch sensitization in AD (40). Pruritus is a common symptom in both AD and keloids. Investigating the relationship between pruritus severity and epidermal IL-33 infiltration may offer a non-invasive method to assess keloid inflammation in the future.

While this study provides significant insights, it has certain limitations. First, the role of JAK1 in IFN-γ-induced IL-33 production was primarily inferred through the use of the JAK1 inhibitor Itacitinib, which has weak affinity for JAK2. Knockdown experiments for JAK1 and JAK2 using shRNA would provide stronger evidence. Second, interactions between secreted IL-33 and ST2 are rapid and transient, with cleaved IL-33 exhibiting diverse recognition sites, particularly in inflammatory environments. Our monoclonal antibody could not capture all forms of IL-33, explaining the subtle changes detected by WB, despite their biological relevance. Future studies will focus on detecting extracellular IL-33 using ELISA or WB and distinguishing nuclear from extracellular IL-33 to further evaluate their respective functions.

Finally, due to the loss of epigenetic features during culture, primary keratinocytes derived from keloid tissues were not used. Instead, HaCaT cells and patient-derived PBMCs were employed to partially model keloid pathology. We hope that the development of stable keratinocyte cell lines in the future will allow us to validate our findings further.

Our study underscores the critical role of epidermal inflammation in keloid formation and maintenance. We highlight the IFN-γ-IL-33 loop as a central mechanism driving epidermal dysfunction and as a potential therapeutic target for keloid treatment.

## Data Availability

The original contributions presented in the study are included in the article/**Supplementary Material**. Further inquiries can be directed to the corresponding authors.

## References

[1] Akaishi S Akimoto M Ogawa R Hyakusoku H. The relationship between keloid growth pattern and stretching tension: visual analysis using the finite element method. Ann Plast surgery. (2008) 60:445–51. doi: 10.1097/SAP.0b013e3181238dd7 18362577

[2] Cohen AJ Nikbakht N Uitto J. Keloid disorder: genetic basis, gene expression profiles, and immunological modulation of the fibrotic processes in the skin. Cold Spring Harb Perspect Biol. (2023) 15:a041245. doi: 10.1101/cshperspect.a041245 36411063 PMC10317059

[3] Ogawa R. Keloid and hypertrophic scars are the result of chronic inflammation in the reticular dermis. Int J Mol Sci. (2017) 18:606. doi: 10.3390/ijms18030606 28287424 PMC5372622

[4] Wang ZC Zhao WY Cao Y Liu YQ Sun Q Shi P. The roles of inflammation in keloid and hypertrophic scars. Front Immunol. (2020) 11:603187. doi: 10.3389/fimmu.2020.603187 33343575 PMC7746641

[5] Bagabir R Byers RJ Chaudhry IH Muller W Paus R Bayat A. Site-specific immunophenotyping of keloid disease demonstrates immune upregulation and the presence of lymphoid aggregates. Br J Dermatol. (2012) 167:1053–66. doi: 10.1111/j.1365-2133.2012.11190.x 23106354

[6] Nangole FW Ouyang K Anzala O Ogengo J Agak GW. Multiple cytokines elevated in patients with keloids: is it an indication of auto-inflammatory disease? J Inflammation Res. (2021) 14:2465–70. doi: 10.2147/JIR.S312091 PMC8203597 34140794

[7] Wu J Del Duca E Espino M Gontzes A Cueto I Zhang N. RNA sequencing keloid transcriptome associates keloids with th2, th1, th17/th22, and JAK3-skewing. Front Immunol. (2020) 11:597741. doi: 10.3389/fimmu.2020.597741 33329590 PMC7719808

[8] Cayrol C Girard JP. Interleukin-33 (IL-33): A nuclear cytokine from the IL-1 family. Immunol Rev. (2018) 281:154–68. doi: 10.1111/imr.2018.281.issue-1 29247993

[9] Liew FY Girard JP Turnquist HR. Interleukin-33 in health and disease. Nat Rev Immunol. (2016) 16:676–89. doi: 10.1038/nri.2016.95 27640624

[10] Wang L Tang J Yang X Zanvit P Cui K Ku WL. TGF-beta induces ST2 and programs ILC2 development. Nat Commun. (2020) 11:35. doi: 10.1038/s41467-019-13734-w 31911623 PMC6946674

[11] Gronberg C Rattik S Tran-Manh C Zhou X Rius Rigau A Li YN. Combined inhibition of IL-1, IL-33 and IL-36 signalling by targeting IL1RAP ameliorates skin and lung fibrosis in preclinical models of systemic sclerosis. Ann Rheum Dis. (2024) 83:1156–68. doi: 10.1136/ard-2023-225158 PMC11420747 38594058

[12] Qiu Z Zhu Z Liu X Chen B Yin H Gu C. A dysregulated sebum-microbial metabolite-IL-33 axis initiates skin inflammation in atopic dermatitis. J Exp Med. (2022) 219:e20212397. doi: 10.1084/jem.20212397 35977109 PMC9375142

[13] Dai X Utsunomiya R Shiraishi K Mori H Muto J Murakami M. Nuclear IL-33 plays an important role in the suppression of FLG, LOR, keratin 1, and keratin 10 by IL-4 and IL-13 in human keratinocytes. J Invest Dermatol. (2021) 141:2646–55 e6. doi: 10.1016/j.jid.2021.04.002 33865911

[14] Ameri AH Moradi Tuchayi S Zaalberg A Park JH Ngo KH Li T. IL-33/regulatory T cell axis triggers the development of a tumor-promoting immune environment in chronic inflammation. Proc Natl Acad Sci U S A. (2019) 116:2646–51. doi: 10.1073/pnas.1815016116 PMC6377481 30696763

[15] Limandjaja GC van den Broek LJ Waaijman T van Veen HA Everts V Monstrey S. Increased epidermal thickness and abnormal epidermal differentiation in keloid scars. Br J Dermatol. (2017) 176:116–26. doi: 10.1111/bjd.2017.176.issue-1 27377288

[16] Chen Z Gao Z Xia L Wang X Lu L Wu X. Dysregulation of DPP4-CXCL12 balance by TGF-beta1/SMAD pathway promotes CXCR4(+) inflammatory cell infiltration in keloid scars. J Inflammation Res. (2021) 14:4169–80. doi: 10.2147/JIR.S326385 PMC8408422 34483675

[17] Chen Z Zhou L Won T Gao Z Wu X Lu L. Characterization of CD45RO(+) memory T lymphocytes in keloid disease. Br J Dermatol. (2018) 178:940–50. doi: 10.1111/bjd.16173 29194570

[18] Guttman-Yassky E Irvine AD Brunner PM Kim BS Boguniewicz M Parmentier J. The role of Janus kinase signaling in the pathology of atopic dermatitis. J Allergy Clin Immunol. (2023) 152:1394–404. doi: 10.1016/j.jaci.2023.07.010 37536511

[19] Dai X Shiraishi K Muto J Utsunomiya R Mori H Murakami M. Nuclear IL-33 plays an important role in IL-31–Mediated downregulation of FLG, keratin 1, and keratin 10 by regulating signal transducer and activator of transcription 3 activation in human keratinocytes. J Invest Dermatol. (2022) 142:136–44 e3. doi: 10.1016/j.jid.2021.05.033 34293350

[20] England E Rees DG Scott IC Carmen S Chan DTY Chaillan Huntington CE. Tozorakimab (MEDI3506): an anti-IL-33 antibody that inhibits IL-33 signalling via ST2 and RAGE/EGFR to reduce inflammation and epithelial dysfunction. Sci Rep. (2023) 13:9825. doi: 10.1038/s41598-023-36642-y 37330528 PMC10276851

[21] Saito N Yoshioka N Abe R Qiao H Fujita Y Hoshina D. Stevens-Johnson syndrome/toxic epidermal necrolysis mouse model generated by using PBMCs and the skin of patients. J Allergy Clin Immunol. (2013) 131:434–41 e1-9. doi: 10.1016/j.jaci.2012.09.014 23111236

[22] Moussion C Ortega N Girard JP. The IL-1-like cytokine IL-33 is constitutively expressed in the nucleus of endothelial cells and epithelial cells *in vivo*: a novel 'alarmin'? PloS One. (2008) 3:e3331. doi: 10.1371/journal.pone.0003331 18836528 PMC2556082

[23] Gajardo Carrasco T Morales RA Perez F Terraza C Yanez L Campos-Mora M. Alarmin' Immunologists: IL-33 as a putative target for modulating T cell-dependent responses. Front Immunol. (2015) 6:232. doi: 10.3389/fimmu.2015.00232 26082774 PMC4451696

[24] Griesenauer B Paczesny S. The ST2/IL-33 axis in immune cells during inflammatory diseases. Front Immunol. (2017) 8:475. doi: 10.3389/fimmu.2017.00475 28484466 PMC5402045

[25] Kotsiou OS Gourgoulianis KI Zarogiannis SG. IL-33/ST2 axis in organ fibrosis. Front Immunol. (2018) 9:2432. doi: 10.3389/fimmu.2018.02432 30405626 PMC6207585

[26] Drake LY Kita H. IL-33: biological properties, functions, and roles in airway disease. Immunol Rev. (2017) 278:173–84. doi: 10.1111/imr.2017.278.issue-1 PMC5492954 28658560

[27] Tominaga M Takamori K. Peripheral itch sensitization in atopic dermatitis. Allergol Int. (2022) 71:265–77. doi: 10.1016/j.alit.2022.04.003 35624035

[28] Kopach P Lockatell V Pickering EM Haskell RE Anderson RD Hasday JD. IFN-γ directly controls IL-33 protein level through a STAT1- and LMP2-dependent mechanism. J Biol Chem. (2014) 289:11829–43. doi: 10.1074/jbc.M113.534396 PMC4002090 24619410

[29] Meephansan J Tsuda H Komine M Tominaga S Ohtsuki M. Regulation of IL-33 expression by IFN-γ and tumor necrosis factor-α in normal human epidermal keratinocytes. J Invest Dermatol. (2012) 132:2593–600. doi: 10.1038/jid.2012.185 22673732

[30] Zhou Y Xu Z Liu Z. Role of IL-33-ST2 pathway in regulating inflammation: current evidence and future perspectives. J Transl Med. (2023) 21:902. doi: 10.1186/s12967-023-04782-4 38082335 PMC10714644

[31] Tong L Corrales RM Chen Z Villarreal AL De Paiva CS Beuerman R. Expression and regulation of cornified envelope proteins in human corneal epithelium. Invest Ophthalmol Vis Sci. (2006) 47:1938–46. doi: 10.1167/iovs.05-1129 PMC2906387 16639001

[32] Kim BE Kim J Goleva E Berdyshev E Lee J Vang KA. Particulate matter causes skin barrier dysfunction. JCI Insight. (2021) 6:e145185. doi: 10.1172/jci.insight.145185 33497363 PMC8021104

[33] Zhang JJ He XC Zhou M Liu QD Xu WZ Yan YJ. Xiao-qing-long-tang ameliorates OVA-induced allergic rhinitis by inhibiting ILC2s through the IL-33/ST2 and JAK/STAT pathways. Phytomedicine. (2023) 119:155012. doi: 10.1016/j.phymed.2023.155012 37586158

[34] Schiering C Krausgruber T Chomka A Frohlich A Adelmann K Wohlfert EA. The alarmin IL-33 promotes regulatory T-cell function in the intestine. Nature. (2014) 513:564–8. doi: 10.1038/nature13577 PMC4339042 25043027

[35] Curren B Ahmed T Howard DR Ashik Ullah M Sebina I Rashid RB. IL-33-induced neutrophilic inflammation and NETosis underlie rhinovirus-triggered exacerbations of asthma. Mucosal Immunol. (2023) 16:671–84. doi: 10.1016/j.mucimm.2023.07.002 37506849

[36] Ung CY Warwick A Onoufriadis A Barker JN Parsons M McGrath JA. Comorbidities of keloid and hypertrophic scars among participants in UK biobank. JAMA Dermatol. (2023) 159:172–81. doi: 10.1001/jamadermatol.2022.5607 PMC9857738 36598763

[37] Lu YY Lu CC Yu WW Zhang L Wang QR Zhang CL. Keloid risk in patients with atopic dermatitis: a nationwide retrospective cohort study in Taiwan. BMJ Open. (2018) 8:e022865. doi: 10.1136/bmjopen-2018-022865 PMC6059319 30021755

[38] Imai Y Yasuda K Sakaguchi Y Haneda T Mizutani H Yoshimoto T. Skin-specific expression of IL-33 activates group 2 innate lymphoid cells and elicits atopic dermatitis-like inflammation in mice. Proc Natl Acad Sci U S A. (2013) 110:13921–6. doi: 10.1073/pnas.1307321110 PMC3752227 23918359

[39] Seltmann J Roesner LM von Hesler FW Wittmann M Werfel T. IL-33 impacts on the skin barrier by downregulating the expression of filaggrin. J Allergy Clin Immunol. (2015) 135:1659–61 e4. doi: 10.1016/j.jaci.2015.01.048 25863977

[40] Gao TC Wang CH Wang YQ Mi WL. IL-33/ST2 signaling in the pathogenesis of chronic pain and itch. Neuroscience. (2023) 529:16–22. doi: 10.1016/j.neuroscience.2023.08.013 37574108

