# Supplementary material for: Keloids and inflammation: the crucial role of IL-33 in epidermal changes
Source: Front Immunol. 2025 Mar 31;16:1514618. doi: 10.3389/fimmu.2025.1514618 (PMC11994421; doi:10.3389/fimmu.2025.1514618)
Supplement: Supplementary file 2 [file Table1.docx]

**Keloids and Inflammation: The Crucial Role of IL-33 in Epidermal Changes**

**ZongAn Chen^#^, YaTing Yang^#^, XiuXia Wang^#^, LingLing Xia, WenBo Wang, XiaoLi Wu*, Zhen Gao*.**

From the Department of Plastic and Reconstructive Surgery, Shanghai Ninth People’s Hospital, Shanghai Jiao Tong University School of Medicine, Shanghai 200025, China.

*****Corresponding author

Shanghai Ninth People's Hospital, Shanghai Jiao Tong University School of Medicine. Shanghai 200011, China.

Telephone number: +86 021-23271699.

Email: [shgaozhen@126.com](mailto:shgaozhen@126.com); wuxiaoli528@126.com

**^#^**These authors contributed equally to this work and should be considered co-first authors.


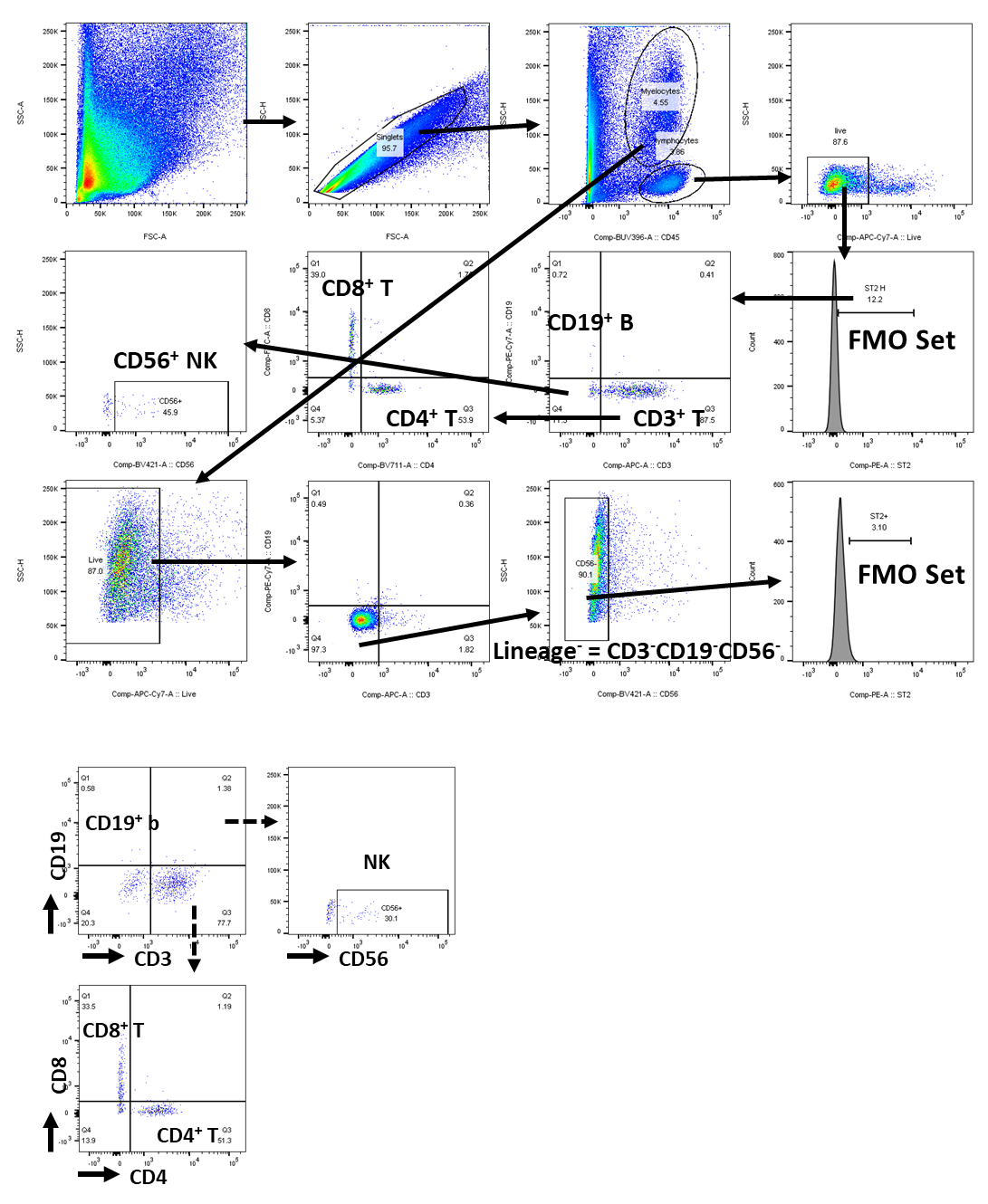


Supplementary Figure 1. Gating Strategy for ST2+ lymphocytes among CD45+ cells in keloid derived cells.


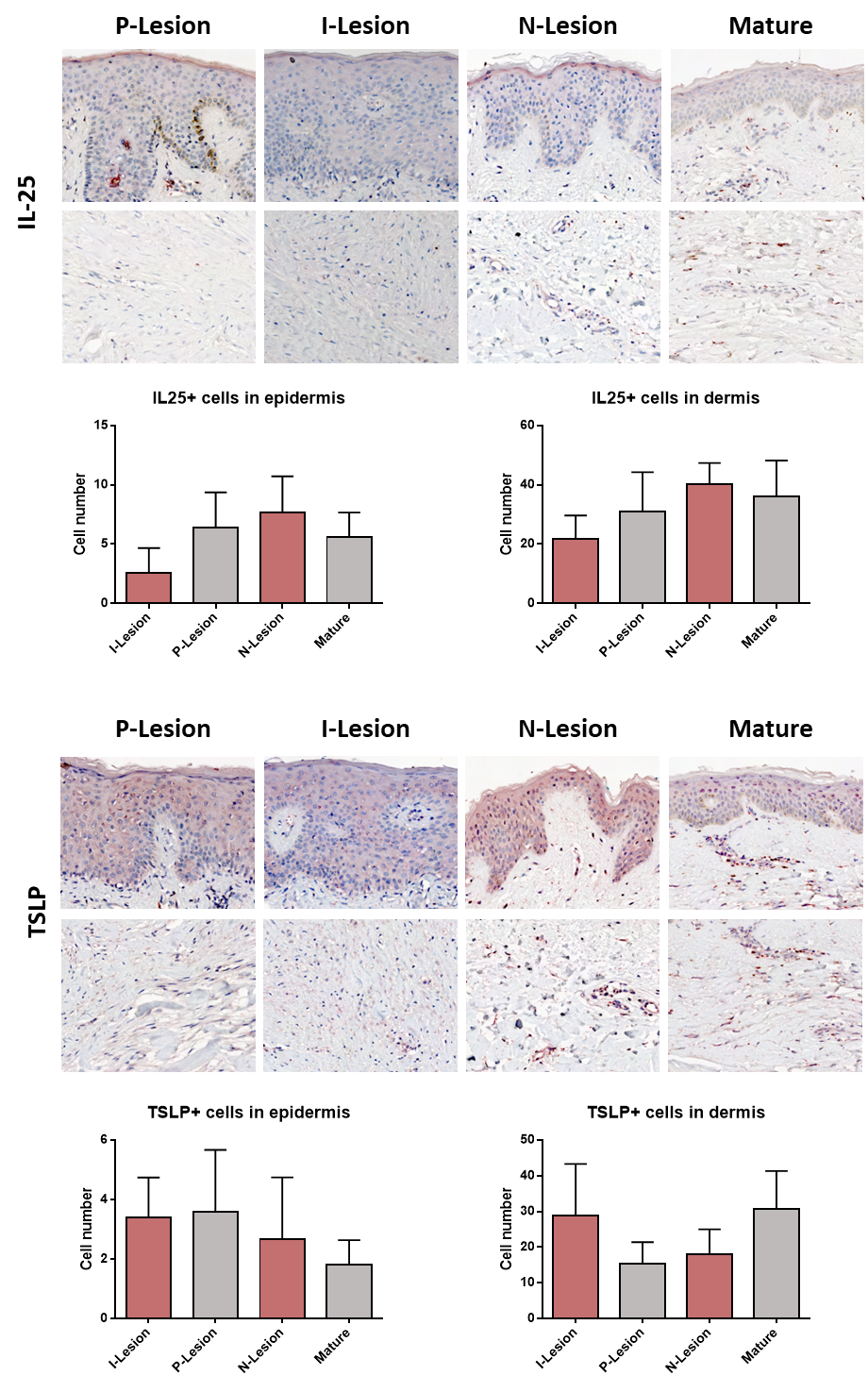


Supplementary Figure 2. IHC analysis of IL25 and TSLP expression in the epidermis and dermis of mature scars and different sites of keloid scars. No statistical significance was observed among all groups.


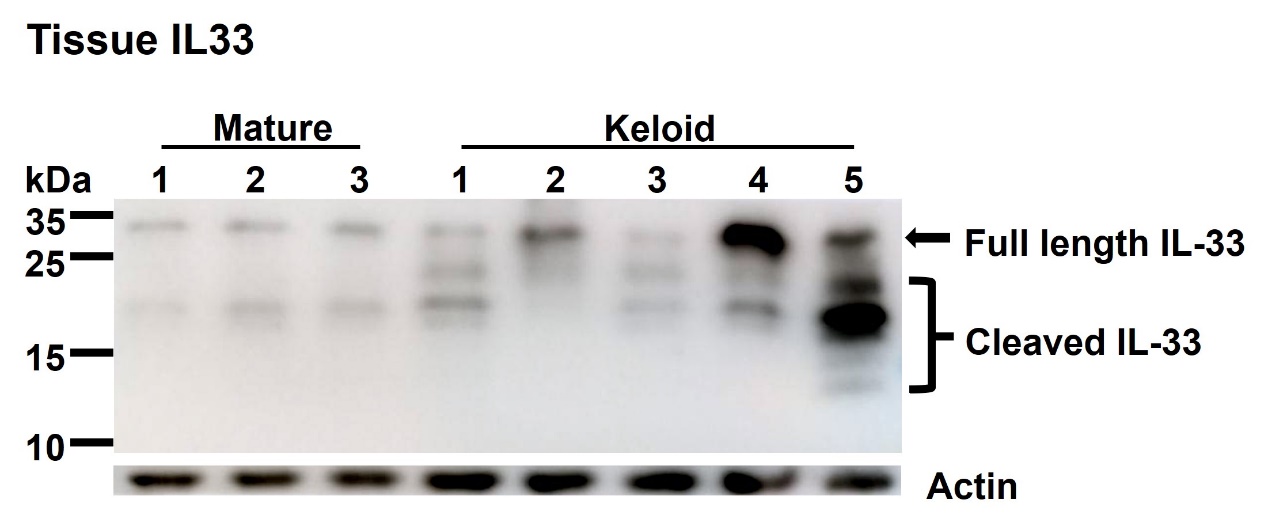


Supplementary Figure 3. WB analysis of cleaved IL-33 and full-length IL-33 in mature scar tissues (n=3) and keloid scar tissues (n=5).


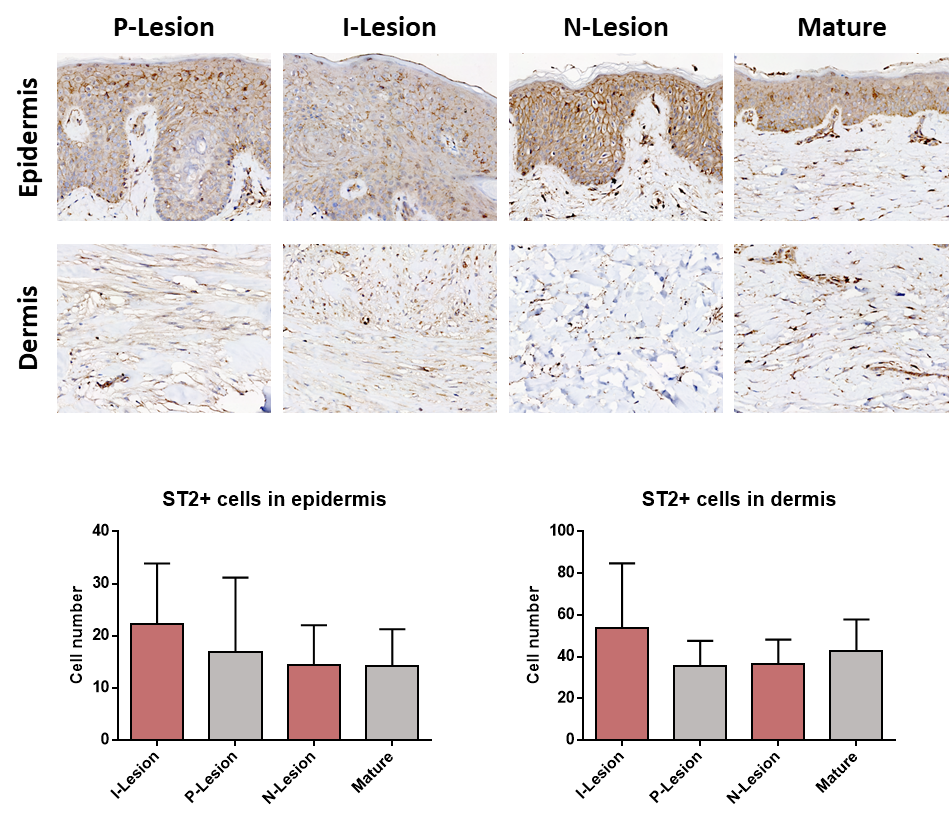


Supplementary Figure 4. IHC analysis of ST2 in the epidermis and dermis of mature scars and different sites of keloid scars. No statistical significance was observed among all groups.


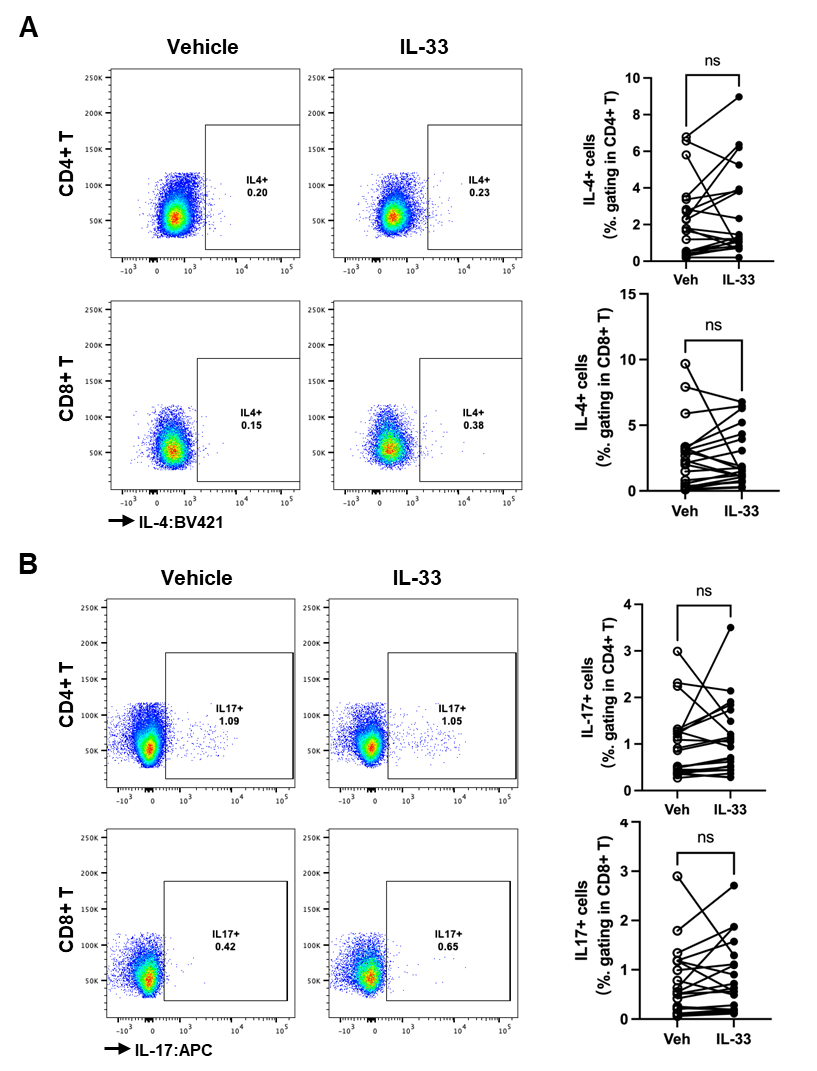


Supplementary Figure 5. Flow cytometric analysis of IL-4(+) (A) and IL-17(+) (B) lymphocytes after pretreated with100ng/mL IL-33 for 24 h. ns represents no significance.


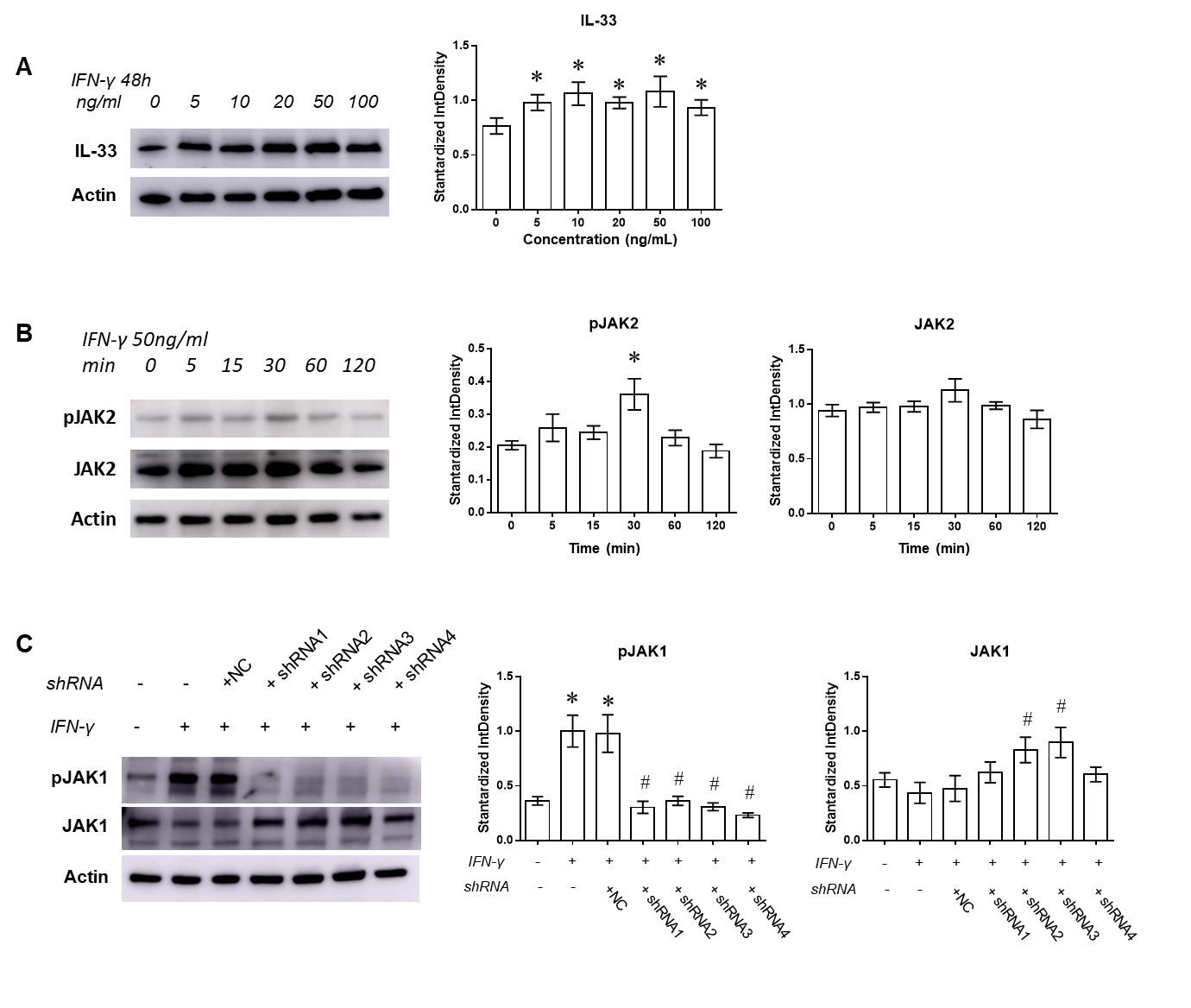


Supplementary Figure 6. WB analysis of HaCaT cells. A. WB plot and statistical analysis of IL-33 in HaCaT cells treated with 0, 5, 10, 20, 50, and 100 ng/mL IFN-γ. *represents P < 0.05 in comparison with cells treated with 0 ng/mL IFN-γ. B. WB plot and statistical analysis of pJAK2 and JAK2 in HaCaT cells treated with 50 ng/mL IFN-γ for 0, 5, 15, 30, 60, and 120 min. C. WB plot and statistical analysis of pJAK1 and JAK1 in HaCaT cells treated with or without shRNA1, shRNA2, shRNA3, and shRNA4 in presence of 50 ng/mL IFN-γ. *represents P < 0.05 in comparison with cells treated without IFN-γ or shRNA. ^#^ represents P < 0.05 in comparison with cells treated with IFN-γ alone. NC refers to negative control.

Supplementary Table 1. Clinical characteristics of volunteers.

| Code | Group | Age | Gender | Site |
| --- | --- | --- | --- | --- |
| K058 | Keloid scar | 23 | F | Chest |
| K059 | Keloid scar | 27 | F | Chest |
| K087 | Keloid scar | 32 | F | Chest |
| K094 | Keloid scar | 40 | F | Abdomen |
| K099 | Keloid scar | 25 | M | Chest |
| K101 | Keloid scar | 25 | F | Back |
| K102 | Keloid scar | 28 | F | Back |
| K105 | Keloid scar | 27 | M | Chest |
| K106 | Keloid scar | 30 | F | Chest |
| K107 | Keloid scar | 31 | F | Chest |
| K111 | Keloid scar | 33 | F | Chest |
| K113 | Keloid scar | 36 | M | Chest |
| M070 | Normal scar | 26 | M | Face |
| M071 | Normal scar | 24 | F | Abdomen |
| M077 | Normal scar | 28 | F | Neck |
| M078 | Normal scar | 33 | M | Limb |
| M079 | Normal scar | 25 | F | Face |
| M083 | Normal scar | 41 | F | Neck |
| M094 | Normal scar | 31 | M | Face |
| M095 | Normal scar | 33 | F | Abdomen |
| M097 | Normal scar | 37 | F | Abdomen |
| M098 | Normal scar | 31 | F | Limb |
| M099 | Normal scar | 32 | F | Abdomen |

Supplementary Table 2. Antibodies used for IHC, IF, or WB in this study.

| Target | Source | Ratio | Reagents | Code No. |
| --- | --- | --- | --- | --- |
| ST2 | Rabbit | 1:1000 (IF) | Atlas Antibodies | HPA007406 |
| IL-33 | Rabbit | 1:500 (IHC)  1:200 (IF)  1:1000 (WB) | Abcam | AB207737 |
| IL-25 | Mouse |  | Bio-Techne | MAB1258-SP |
| TSLP | Rabbit | 1:200 (IHC) | Proteintech | 13778-1-AP |
| Keratin10 | Rabbit | 1:1000 (IF) | Abcam | ab76318 |
| Keratin14 | Mouse | 1:500 (IF) | Abcam | ab7800 |
| Cytokeratin1 | Mouse | 1:1000 (WB) | Santa | sc-376224 |
| Filaggrin | Mouse | 1:1000 (WB) | Santa | sc-66192 |
| involucrin | Mouse | 1:1000 (WB) | Santa | sc-21748 |
| pJAK1 | Rabbit | 1:1000 (WB) | CST | 74129S |
| JAK1 | Rabbit | 1:1000 (WB) | CST | 3344T |
| pSTAT1 | Rabbit | 1:1000 (WB) | CST | 7649T |
| STAT1 | Mouse | 1:1000 (WB) | Santa | sc-464 |
| pJAK2 | Rabbit | 1:1000 (WB) | CST | 8082 |
| JAK2 | Rabbit | 1:1000 (WB) | CST | 3230 |

Supplementary Table 3. Antibodies used for FCM in this study.

| Target | Source | Clone | Conjugate | Catalog No. | Reagents |
| --- | --- | --- | --- | --- | --- |
| CD45 | Mouse | HI30 | BUV395 | 563792 | BD Bioscience |
| CD3 | Mouse | SK7 | PerCP | 344814 | Biolegend |
| CD19 | Mouse | SJ25C1 | PE-Cy7 | 557835 | BD Bioscience |
| CD56 | Mouse | NCAM16.2 | BV421 | 562751 | BD Bioscience |
| CD4 | Mouse | RPA-T4 | FITC | 555346 | BD Bioscience |
| CD8 | Mouse | RPA-T8 | PerCP-Cy^TM^5.5 | 560662 | BD Bioscience |
| ST2 | Mouse | A16008J | PE | 379404 | Biolegend |
